# *Streptococcus pneumoniae* TIGR4 Flavodoxin: Structural and Biophysical Characterization of a Novel Drug Target

**DOI:** 10.1371/journal.pone.0161020

**Published:** 2016-09-20

**Authors:** Ángela Rodríguez-Cárdenas, Adriana L. Rojas, María Conde-Giménez, Adrián Velázquez-Campoy, Ramón Hurtado-Guerrero, Javier Sancho

**Affiliations:** 1 Department of Biochemistry and Molecular and Cell Biology, University of Zaragoza, Zaragoza, Spain; 2 Institute of Biocomputation and Physics of Complex Systems (BIFI), University of Zaragoza, Joint Unit IQFR-CSIC-BIFI, Joint Unit EEAD-CSIC-BIFI, Mariano Esquillor s/n, Campus Rio Ebro, Edificio I+D, Zaragoza, Spain; 3 Aragon Health Research Institute (IIS Aragón), University of Zaragoza, Zaragoza, Spain; 4 Structural Biology Unit, CIC bioGUNE, Bizkaia Technology Park, Derio, Spain; 5 Fundación ARAID, Government of Aragón, Zaragoza, Spain; University of Queensland, AUSTRALIA

## Abstract

*Streptococcus pneumoniae* (*Sp*) strain TIGR4 is a virulent, encapsulated serotype that causes bacteremia, otitis media, meningitis and pneumonia. Increased bacterial resistance and limited efficacy of the available vaccine to some serotypes complicate the treatment of diseases associated to this microorganism. Flavodoxins are bacterial proteins involved in several important metabolic pathways. The *Sp flavodoxin* (*Spfld*) gene was recently reported to be essential for the establishment of meningitis in a rat model, which makes *Sp*Fld a potential drug target. To facilitate future pharmacological studies, we have cloned and expressed *Sp*Fld in *E*. *coli* and we have performed an extensive structural and biochemical characterization of both the apo form and its active complex with the FMN cofactor. *Sp*Fld is a short-chain flavodoxin containing 146 residues. Unlike the well-characterized long-chain apoflavodoxins, the *Sp* apoprotein displays a simple two-state thermal unfolding equilibrium and binds FMN with moderate affinity. The X-ray structures of the apo and holo forms of *Sp*Fld differ at the FMN binding site, where substantial rearrangement of residues at the 91–100 loop occurs to permit cofactor binding. This work will set up the basis for future studies aiming at discovering new potential drugs to treat *S*. *pneumoniae* diseases through the inhibition of *Sp*Fld.

## Introduction

*Streptococcus pneumoniae* is a gram-positive bacterium that causes pneumonia, meningitis, otitis media, acute sinusitis, septicemia, and conjunctivitis, among several other diseases [1–4]. The World Health Organization (WHO) considers *Streptococcus pneumoni*ae a major health problem due to the elevated mortality of some of the diseases associated to this pathogen. It is estimated that close to 500,000 children under 5 years died in 2008 as a consequence of pneumococcal infections [5]. More specifically, pneumococcal meningitis causes death in around 25% of cases, plus neurological diseases in half of the survivors [6]. The emerging resistance of *S*. *pneumoniae* to established antibiotics [7], the fact that current polysaccharide-related vaccines are only protective to certain infective serotypes, and the fluctuation of the prevalence of some pneumococcal serotypes over time due to selection forces (i.e.: 7vPCV vaccine) or serotype replacement [8] advice for the identification of new *S*. *pneumoniae* targets, common to the different serotypes, that could be used to develop novel antibiotics to specifically combat *S*. *pneumoniae* infections.

In a recent work [9], several *Sp* TIGR4 genes [10] were identified as essential for *S*. *pneumoniae* infection during experimental meningitis in rats. One of them is the gene encoding flavodoxin (*Spfld*), a protein that has been shown to be essential for several other bacteria (http://tubic.tju.edu.cn/deg/) [11], including *Escherichia coli* [12, 13], *Salmonella enterica* [14], *Haemophilus influenzae* [15], *Vibrio cholerae* [16], and *Helicobacter pylori* [17]. Flavodoxins are electron transfer proteins that participate in a variety of reactions in prokaryotes and in some unicellular algae [18]. In *Azobacter vinelandii*, Fld participates in the reduction of nitrate [19], in *E*. *coli* it mediates the activation of different enzymes such as anaerobic ribonucleotide reductase [20], cobalamin-dependent methionine synthase [21], biotin synthase [22] and pyruvate formate-lyase [23]. In some organisms, such as *S*. *pneumoniae*, the importance of flavodoxin has been tentatively associated to its participation in methionine synthesis [9]. The identification of flavodoxin as an essential protein for *S*. *pneumoniae* infectivity and the fact that flavodoxin is not present in humans open the possibility of using it as a target for the development of novel and specific antimicrobials against *S*. *pneumoniae*. Related to this potential use of flavodoxins as drug targets [24] the flavodoxin from *Helicobacter pylori* (*HpFld*) has been subjected to detailed functional [25], structural [26] and biophysical [27–30] analyses that have facilitated the discovery of novel specific inhibitors with bactericidal properties [31, 32]. In contrast to this wealth of information on *Hp*Fld, very little is known of *Sp*Fld. According to its gene sequence translation [9], it contains 147 residues and very likely belongs to the so-called short-chain flavodoxins [18] characterized by the lack of a 20-residue loop that, in the long-chain flavodoxins, appears intercalated in the fifth β-strand. Hence, *SpFld* shows a higher sequence identity with other short-chain flavodoxins, such as *Streptococcus pyrogenes* (76%) or *Desulfovibrio desulfuricans* (45,3%), than with long-chain flavodoxins, such as those from *Helicobacter pylori* (25,7%), *Anabaena* PCC7119 (22,4%) or *Escherichia coli* (25,7%). The more detailed flavodoxin molecular and structural studies have been devoted to a few long-chain flavodoxins, especially those from *Anabaena* PCC7119 [33–35], *Azobobacter vinelandii* [36] and *Helicobacter pylori* [17, 28]. The lack of information about *Sp*Fld can hardly be alleviated by resourcing to the limited data available for other short-chain flavodoxins. In this work we provide a detailed biophysical characterization of *Sp*Fld and report the x-ray structures of the apo and holo forms, the conformational stability of the protein and the thermodynamic characterization of the cofactor binding equilibrium. These studies provide the target characterization needed to pursue the rational discovery of specific inhibitors against *Sp*Fld. In addition, they will contribute to understand better the specific structural and thermodynamic characteristics of short-chain flavodoxins.

## Results and Discussion

### Spectroscopic properties of the holo and apo *Sp*Fld forms

**Fig 1:**
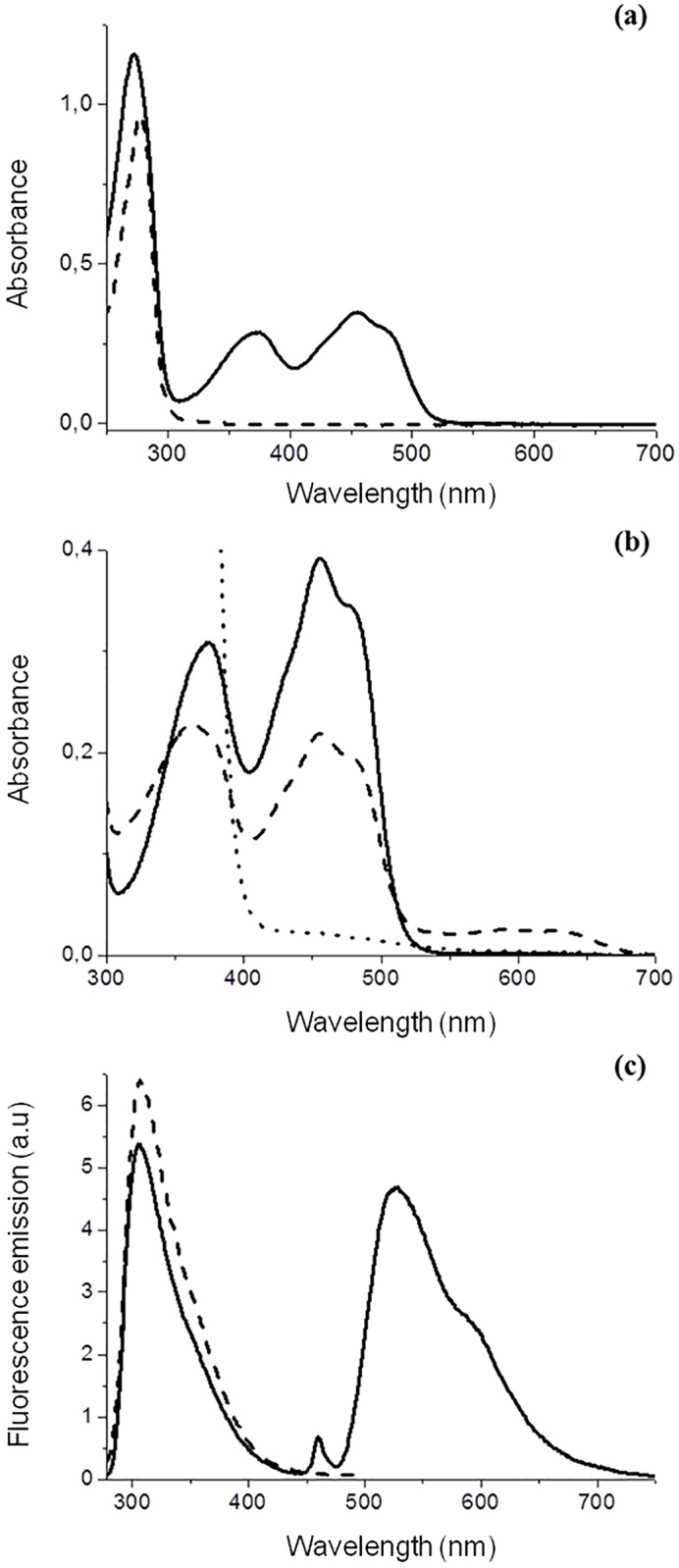
*S*. *pneumoniae* flavodoxin absorption and fluorescence spectra. **(a)** UV/VIS absorbance of 39 μM holo (solid line) and 134 μM apoflavodoxin (dashed line) in 150 mM NaCl, 100 mM MES, pH 6.5. **(b)** Spectra related to three redox states of 43 μM *Sp*Fld in 150 mM NaCl, 100 mM MES, pH 6.5 at 10°C: Oxidized (solid line), semiquinone (dashed line) with absorption maxima at 592 nm and reduced (dotted line). **(c)** Fluorescence emission spectra of 20 μM *Sp*Fld holoprotein (solid line) and 20 μM apoprotein (dashed line), in 150 mM NaCl, 100 mM MES, pH 6.5, 10°C.

The UV-visible absorption spectrum of purified *Sp*Fld (Fig 1a) shows the typical hallmarks of flavin binding proteins, with two sizable absorption peaks at 371 and 456 nm, a pronounced shoulder at 480 nm, and no absorption beyond 550 nm, indicative of a completely oxidized flavin cofactor. The very low 271 nm/456 nm absorbance ratio of 3.3 is consistent with the absence of tryptophan residues and the presence of only five tyrosine residues in its sequence. The UV-visible spectrum of apo *Sp*Fld shows a single peak with maximal absorbance at 277 nm (Fig 1a) and a completely flat spectrum beyond 350 nm, indicative of both a full removal of the cofactor and a lack of protein aggregation phenomena. The apoflavodoxin molar extinction coefficient at 277 nm in 150 mM NaCl, 100 mM MES, pH 6.5, determined as described [37], was 7.2 mM^-1^cm^-1^. The extinction coefficient at 456 nm (λ_max_) of the fully oxidized holoprotein in the same buffer, determined as described [38], was 9.1 mM^-1^cm^-1^. To observe *Sp*Fld reduced species, a partial FMN reduction in pseudo-anaerobic conditions was done with sodium dithionite (Fig 1b). The stabilized semiquinone exhibits a maximum at 592 nm. The low accumulation of semiquinone observed suggests (assuming a typical FMN semiquinone extinction coefficient around 5.0 mM^-1^ cm^-1^ at that wavelength) that the E_ox/sq_ redox potential could be slightly more negative than the E_sq/hq_ one. Most of the flavin signal (95% at λ_max_) is lost in the reduced form. The fluorescence emission spectra of holo *Sp*Fld (Fig 1c) displays characteristic peaks of tyrosine residues and of the FMN cofactor. On the other hand, the apoprotein shows a single maximum of emission at 310 nm, as expected from its amino acid composition.

**Fig 2:**
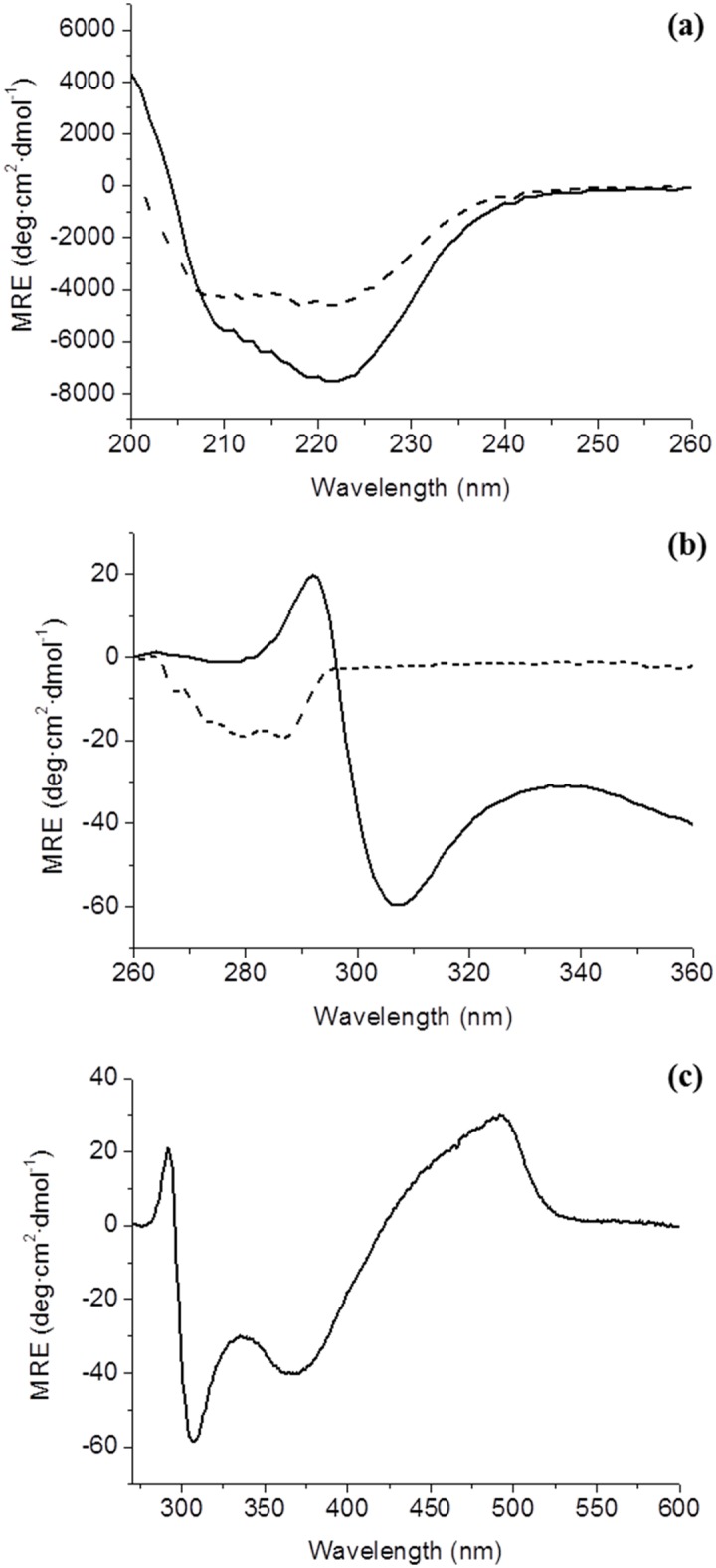
CD spectra of holo and apo *Sp*Fld in 150 mM NaCl, 100 mM MES, pH 6.5, 4°C. **(a)** Far-CD spectra of 80 μM of holo and apo *Sp*Fld (continuous and dashed lines respectively) **(b)** Near-CD spectra of 80 μM of holo (continuous line) and apo (dashed line) *Sp*Fld. **(c)** Visible-CD spectrum of 80 μM of holoprotein.

The far-UV CD spectra of the holo and apoproteins (Fig 2a) indicate that, in solution, the secondary structure content of the apo form is lower than that of the holoprotein. In contrast, in the crystal structures (see below) the two forms appear to display very similar secondary structure contents. The near-UV CD spectra of apo *Sp*Fld (Fig 2b) is characterized by a single negative peak at 280 nm that must arise from the tyrosine residues in the protein. The presence of a peak in this spectral region, which is characteristic of well-folded proteins [39], indicates that, in solution, at least one tyrosine residue is located in a structurally well-defined region. On the other hand, the near-UV CD spectrum of holoprotein (Fig 2b and 2c) is more complex and reveals several contributions of the FMN cofactor that also arise as a consequence of its interaction with a well-structured protein moiety [40]. The FMN-protein interaction gives rise to prominent peaks in the visible region at the wavelengths of maximal absorbance of the FMN cofactor. The CD data together suggests that both the apo and holo forms display well defined tertiary structures in solution, but suggests that the apo form overall secondary structure content may be lower than that observed in the crystal structure (see below). The near-UV CD spectra and the fluorescence emission spectra of apo *Sp*Fld are different from those of *Anabaena* and *Hp* flavodoxins, which are dominated by positive peaks in the near-UV CD and by longer fluorescence emission wavelengths characteristic of tryptophan containing proteins. *Sp*Fld thus displays a specific spectroscopic identity that allows it to be distinguished from the aforementioned long-chain flavodoxins based on simple fluorescence and CD spectra.

### *Sp*Fld thermal stability

**Fig 3:**
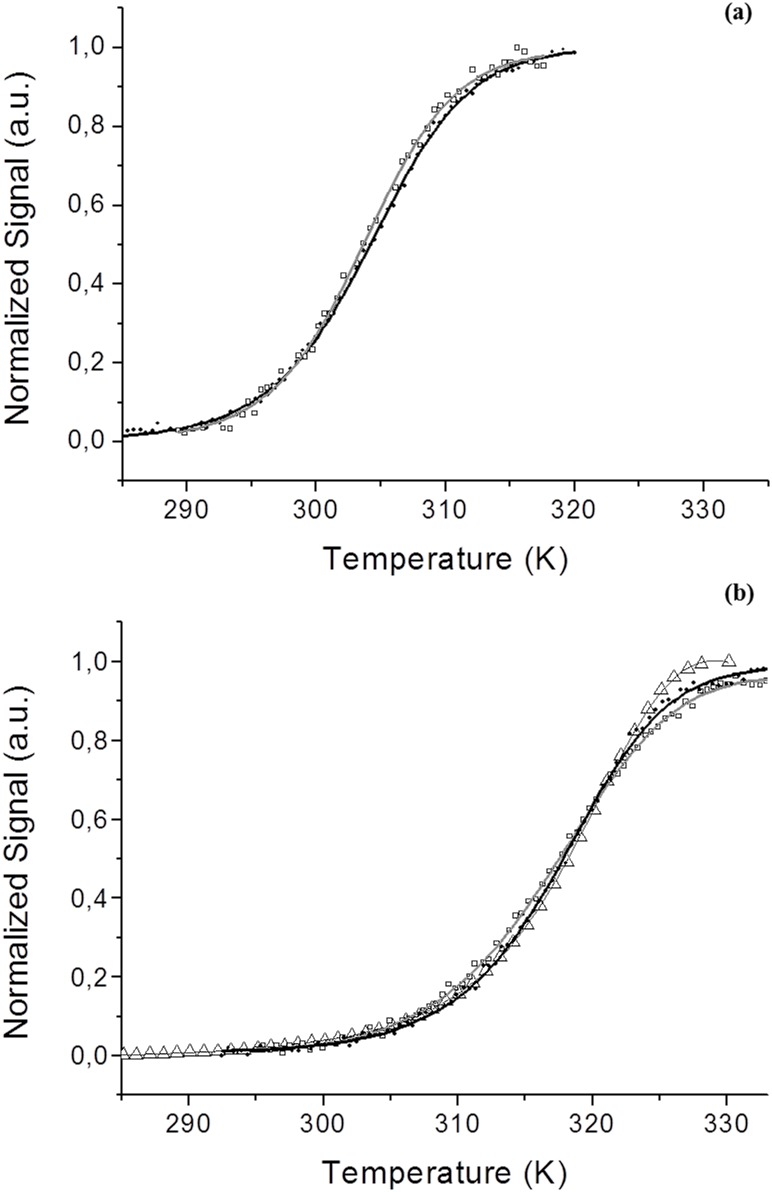
Thermal unfolding curves monitored spectroscopically in 150 mM NaCl, 100 mM MES pH 6.5. **(a)** (80 μM) holo *Sp*Fld by Fluorescence (open triangles, gray line), far-UV CD (black circles, black line) and near-UV CD (open squares, gray line). **(b)** (80 μM) apo *Sp*Fld by far-UV CD (black circles, black line) and near-UV CD (open squares, gray line).

The stability of holo and apo *Sp*Fld has been determined from thermal unfolding curves. The far UV-CD and near-UV CD thermal unfolding curves of the apo *Sp*Fld, roughly normalized to signal spans from approximately 0 to 1, are shown in Fig 3a. Their fair superposition suggests that the apoprotein follows a two-state thermal unfolding mechanism. The individual fits of the two curves to two-state equations (eqs 1 and 2) give similar temperatures of mid denaturation (around 30°C) and similar enthalpy changes (around 50 kcal mol^-1^). Their global analysis confirms the two-state model and provides a *T*_*m*_ of 30.8 ± 2°C and an enthalpy change of 41 ± 5 kcal mol^-1^ (Table 1).

**Table 1:** Thermal stability parameters for holo and apo *Sp*Fld.

		Holo Flavodoxin^a^					Apo Flavodoxin^a^		
	Far-UV CD	Near-UV CD	Fluoresc.	Global fit^b^	DSC	Far-UV CD	Near-UV CD	Global fit^b^	DSC
***T***_***m***_	44.7±0.4	43.7±1	44.5±0.3	44.6±0.2	46.7±0.5	30.3±0.1	30.4±0.8	30.8±2	34.2±0.5
(°C)									
**Δ*H***_**m**_	52±2	38±3	65.8±0.1	51±1	61±2	51±1	50.5±0.6	41±5	55±2
(kcal mol^-1^)									

^a^ In 150 mM NaCl, 100 mM MES, pH 6.5.

^b^ Parameters obtained through global analysis of the spectroscopic curves using a two-state unfolding model.

The thermal unfolding of holo SpFld was monitored by far-UV CD, near-UV CD and FMN fluorescence. The unfolding curves, roughly normalized to signal spans from zero to one (Fig 3b), can also be superimposed, suggesting a simple two-state unfolding equilibrium for the holoprotein [41]. Their individual fitting to a two-state equation provide *T*_*m*_ values around 44.5°C and enthalpy changes around 50 kcal mol^-1^. The global analysis of the three curves gave rise to a *T*_*m*_ of 44.6 ± 0.2°C and an enthalpy change of 51±1 kcal mol^-1^ (Table 1), similar to the values obtained in the individual fits. The fact that the *T*_*m*_ of the holoflavodoxin is higher than that of the apoprotein is the expected behavior for any protein whose native conformation binds a ligand, in this case the FMN cofactor, as it has been discussed before in the context of the apoflavodoxin/FMN equilibrium in other species [28, 30, 42].

**Fig 4:**
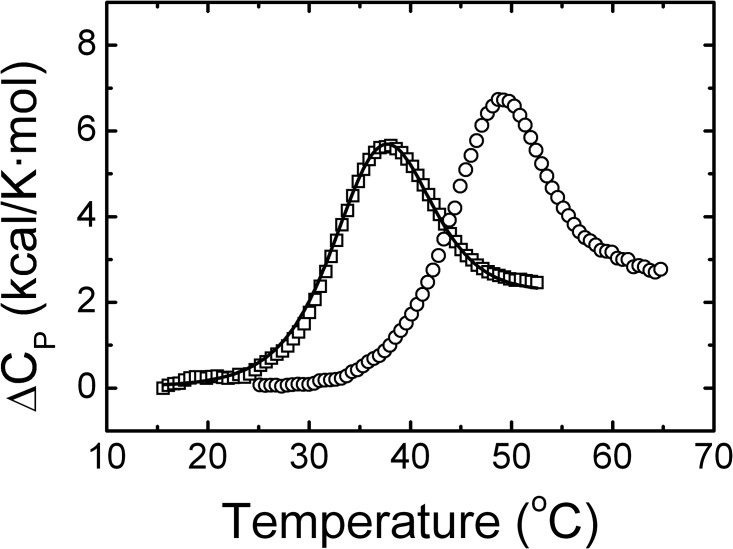
DSC analysis in the absence of FMN (open squares) or in the presence of 40 μM of FMN (open circles) in 150 mM NaCl, 100 mM MES, pH 6.5. The continuous line is the non-linear fitting curve obtained by using a two-state model for the unfolding of apoFld.

The thermal unfolding of the holo and apo *Sp*Fld has also been investigated by DSC (Fig 4). Analysis of the thermograms of either form of the protein confirms that the unfolding equilibrium is two-state with only one cooperative transition. The calorimetric *T*_*m*_ and Δ*H* values are similar to those derived from the spectroscopic analysis (Table 1) and confirm that the holoprotein is more stable than the apoprotein.

Although little is known about the stability of short-chain flavodoxins, the stability of long-chain ones has been studied in detail [18], specially using *Anabaena PCC 7119* flavodoxin, as a model. In long-chain flavodoxins, the thermal unfolding of the apoform is at least three-state (four-state in *H*. *pylori* [28]) because a partly unfolded intermediate conformation accumulates as the temperature is raised before the protein becomes fully unfolded. The structure of this major thermal intermediate has been determined at atomic resolution [43]. Roughly, two-thirds of it displays a native conformation while the other third, encompassing the long loop of long-chain flavodoxins and neighboring residues, is unfolded. In addition, protein engineering experiments have shown that removal of the long loop gives rise to a shortened well-folded *Anabaena* apoflavodoxin, which behaves as two-state towards thermal unfolding [44]. Therefore, it is well established that the equilibrium thermal intermediate of long-chain flavodoxins is associated to the autonomous unfolding of the long loop. Our spectroscopic and calorimetric analysis of the short-chain *Sp* apoflavodoxin thermal unfolding indicates that no thermal intermediate accumulates, which suggests that short-chain apoflavodoxins may generally follow a simple two-state equilibrium unfolding mechanism.

Unlike in previous studies in our group on the stability of other flavodoxins, we have used here a slightly acidic buffer and a higher ionic strength because, otherwise, the conformational stability of *Sp* apoflavodoxin is significantly lower, and it is difficult to determine accurately. In fact, at the conditions typically used to characterize *Anabaena* or *Hp* apoflavodoxins (i.e. pH 7 and around 20 mM ionic strength), *Sp* apoflavodoxin appears to be unfolded (not shown). This lower stability is unlikely related to the absence of a long loop in the short-chain *SpFld*. Our previous analysis of the influence of the *Anabaena* PCC 7119 long loop in the conformational stability of that long-chain flavodoxin [44] showed that removal of the long loop hardly modified the conformational stability of the protein. The low stability of *SpFld* in low ionic strength conditions is more likely related to its peculiar amino acid composition. In fact, the progressively decreasing conformational stability of the *H*. *pylori*, *Anabaena PCC 7119* and *Sp* apoflavodoxins at neutral pH and low ionic strength correlates with their increasing relative content of acidic residues (27/164, 31/169 and 38/148, respectively) that gives rise to an increasingly large excess of negative charge at pH 7 (approximately -12, -17 and -28, respectively). This interpretation is in agreement with the fact that, in the *Anabaena* protein, more stable variants have been engineered by reducing the net negative charge of the apoprotein [45, 46].

### *Sp*Fld interaction with FMN

**Fig 5:**
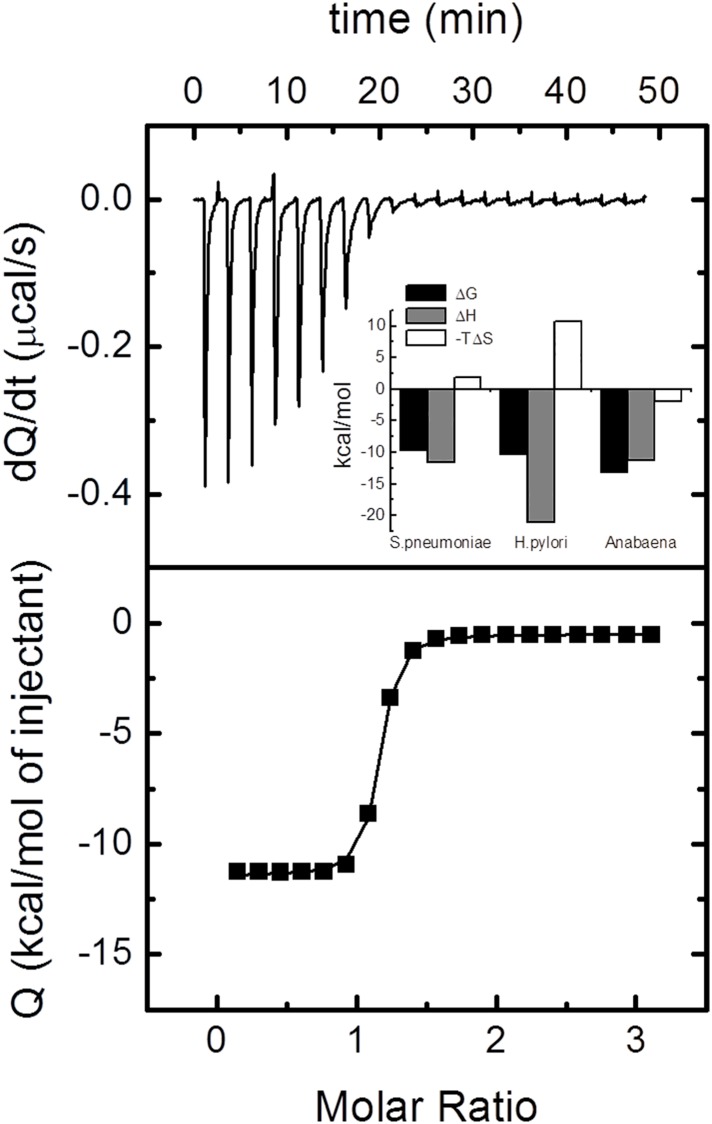
Titration of 20 μM Apo *Sp*Fld with 300 μM FMN at 25°C in 150 mM NaCl, 100 mM MES, pH 6.5. The upper panel shows the thermogram (thermal power as function of time) and the lower panel shows the binding isotherm (normalized heat as a function of the molar ratio). The continuous line is the non-linear fitting curve obtained using a model considering a single ligand binding site. The inset in the upper panel shows a comparison of the thermodynamic binding profiles obtained by ITC for FMN interacting with apoflavodoxins from *S*. *pneumoniae*, *Helicobacter pylori*, and *Anabaena* PCC7119: Δ*G* (black bars) Δ*H* (gray bars) -TΔ*S* (white bars). Data for the *H*. *pylori* and *Anabaena* complexes come from reference [28]

The higher thermostability of *Sp* holoflavodoxin compared to that of the apo form is a consequence of the favorable interactions established between the apoprotein and the cofactor. To quantitate this effect, the interaction between the apoprotein and the FMN cofactor was studied by isothermal titration calorimetry at 15, 20 and 25°C (Fig 5 and Table 2). In addition, buffer-independent binding parameters were determined by using buffers with different ionization enthalpies (Table 2). As observed for other flavodoxins, FMN binding is markedly exothermic. The overall binding process is enthalpically driven, with a small unfavorable entropic contribution. This suggests a considerable conformational entropy loss that is not compensated by the desolvation entropy. A large part of the hydrophobic FMN moiety (isoalloxazine ring) does not become buried inside the binding site; moreover, the polar region of FMN strongly interacts with amino acid residues in the active site and becomes completely buried upon binding. The dissociation constant at 25°C is 83 nM, not far from that of *Helicobacter pylori* flavodoxin (27 nM), but two orders of magnitude higher than that of *Anabaena* flavodoxin (0.26 nM). As previously observed for other flavodoxins, there is no significant exchange of protons coupled to FMN binding (Table 2). The binding data obtained at the three temperatures indicated allows estimating a binding heat capacity change of -0.5 kcal K^-1^·mol^-1^, similar to that of other flavodoxins.

**Table 2:** Apoflavodoxin/FMN binding energetics.

	*S*. *pneumoniae*^b^	*H*. *pylori*^a^	*Anabaena PCC 7119*^a^
**K**_**d**_ (nM)	83±9	27±6	0.26±0.06
**Δ*G*** (kcal mol^-1^)	-9.7±0.1	-10.3±0.1	-13.1±0.1
**Δ*H***_**0**_ (kcal mol^-1^)	-11.6±0.3^c^	-21.0±0.3	-11.2±0.2
**-TΔ*S*** (kcal mol^-1^)	1.9±0.3	10.7±0.3	-1.9±0.2
**Δ*C***_***p***_ (kcal kmol^-1^)	-0.5±0.1^d^	-0.60±0.03	-0.60±0.02
***n***_**H**_	0.1±0.2^c^	0.1±0.2	0.1±0.2

^a^ In 50 mM MOPS, pH 7.0 [28]

^b^ In 100 mM MES, 150 mM NaCl, pH 6.5

^c^ Δ*H*_0_ and *n*_H_ were estimated by linear regression (see Methods).

^d^ Estimated as the slope of Δ*H* vs T from experiments at 15, 20 and 25°C

### X-ray structures of the apo and holo forms of *Sp*Fld

**Fig 6:**
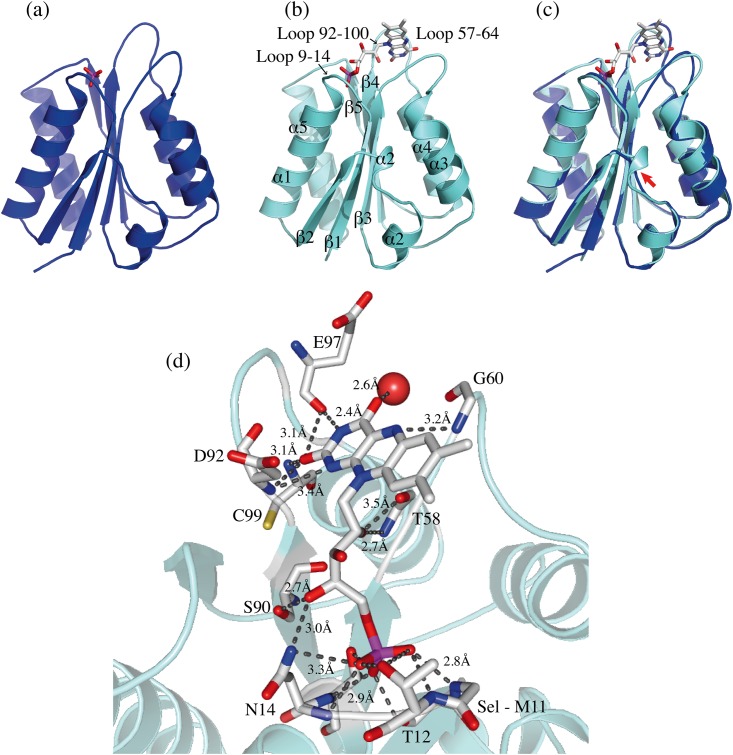
Ribbon structure representation of apo S*p*Fl in blue (a) and holo S*p*Fld in cyan (b). Changes in the key FMN binding loops indicated in (b) can be observed in the structural superimposition of the holo (cyan) and apo (blue) flavodoxins shown in (c). Also in (c), a red arrow points to a short 3_10_ helix formed in the holoprotein. The FMN-*Sp*Fld hydrogen bonding interactions are highlighted in (d).

**Fig 7:**
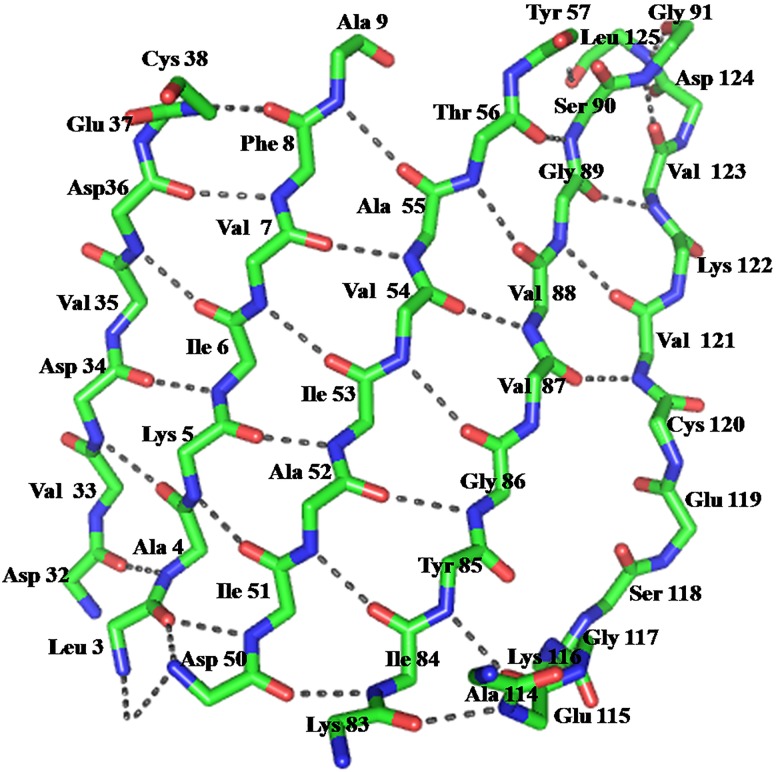
Stick representation of the 5-stranded β-sheet of *Sp*Fld showing a characteristic bulge in strand 5, which has been attributed [44] to originate in evolutionary splicing of two preexisting short strands, 5a and 5b that, in long-chain flavodoxins, are connected by their characteristic long loop.

To understand the molecular basis of the interaction of the FMN cofactor with the protein moiety and to provide a structural frame for the identification and characterization of *Sp*Fld inhibitors, we have solved the crystal structures of the *Sp*Fld in the apo form (Fig 6a) and in complex with FMN (Fig 6b and S1 Fig), at 2.18 and 1.69 Å, respectively (Table 3). Either structure shows the archetypical α/β sandwich flavodoxin fold (5 α-helices that pack against a five-stranded β-sheet with a 21345 topology, forming an α/β/α sandwich). It has been reported that some short-chain flavodoxins contain a bulge in the fifth beta strand that has been interpreted as reminiscent of an evolutionary event where the long loop was lost from long-chain flavodoxins to give rise to short-chain flavodoxins [44]. This unique bulge is also found in the fifth beta strand of *Sp* flavodoxin, unlike in any of the other four strands (Fig 7). Overall, the holo (note that the holo form contains selenomethionine (SeMet) residues) and apo *Sp*Fld structures are similar with an r.m.s.d of 0.8 Å. The main differences between the two structures are traced to two of the three FMN binding loops encompassing residues 10–14 (phosphate loop), 57–66 (50’ loop) and 92–100 (90’ loop), the latter two sandwiching the isoalloxazine moiety. A further noticeable difference is the presence of a small 3_10_ helix stretch at residues 38–40 in the holoprotein that is absent in the apo form (Fig 6c).

**Table 3:** Data collection and refinement statistics of holo and apo *Sp*Fld crystallographic structures.

	*Sp*Fld*-*apo	*Sp*Fld*-*FMN
		(SeMet form)
**Space group**	P2_1_2_1_2_1_	H3
**Wavelength (Å)**	0.92	0.976
**Resolution (Å)**	20–1.60	37.00–2.07
	(1.69–1.60)^a^	(2.18–2.07)
**N. of molecules in Asymmetric unit**	1	2
**Cell dimensions (Å)**	*a = * 29.87	*a = * 101.68
	*b = * 60.71	*b* = 101.68
	*c = * 61.80	*c* = 68.28
**Unique reflections**	15310	15961
**Completeness**	99.2 (99.5)	99.2 (99.6)
***R***_**sym**_	0.102 (0.703)	0.094 (0.421)
***I*/σ(*I*)**	13.9 (2.7)	18.9 (6.7)
**Mn(I) half-set correlation CC(1/2)**	0.998 (0.810)	0.999 (0.934)
**Redundancy**	9.9 (9.6)	16.0 (16.1)
**Alpha_twin**		0.478
***R***_**work**_**/*R***_**free**_	0.204/0.228	0.187/0.228
**RMSD from ideal geometry, bonds (Å)**	0.007	0.017
**RMSD from ideal geometry, angles (°)**	1.448	2.047
**<*B*> overall (Å**^**2**^**)**	15.32	39.22
**<*B*> FMN (Å**^**2**^**)**	-	32.90
**<*B*> solvent (Å**^**2**^**)**	36.28	36.58
**<*B*> phosphate (Å**^**2**^**)**	11.97	-
**Ramachandran plot:**		
**Most favoured (%)**	97.90	96.49
**Additionally allowed (%)**	2.10	3.51
**Generously allowed (%)**		

^**a**^ Values in parentheses refer to the highest resolution shell. Ramachandran plot statistics were determined with PROCHECK, ref. [63].

Similar secondary structure induction due to FMN interaction is also observed in the crystal structures of holo and apo forms of short-chain flavodoxin MioC of *E*. *coli* [47]. The extent of the structural changes brought about by FMN binding in other flavodoxins of known structure has been analysed using DaliLite [48]. The larger r.m.s.d. deviation between the holo and apo forms was found for MioC Flavodoxin (2,6 Å), followed by *Anabaena* and *Sp* (0.8 Å), and then *H*. *pylori* (0.5 Å). Those data show a high preservation of the backbone structure upon FMN binding and indicate that segmental movements are essentially confined to the known dynamic loops of the protein; 50´s-Loop and 90´s-Loop. Smaller changes are observed in the phosphate binding loop upon FMN binding because, in the different apoproteins studied, this loop always carries bound anions: a phosphate anion in *Sp* apoflavodoxin, a chloride anion in *Helicobacter pylori* [26], or a sulfate anion in *Anabaena* [49] (S2 Fig).

**Fig 8:**
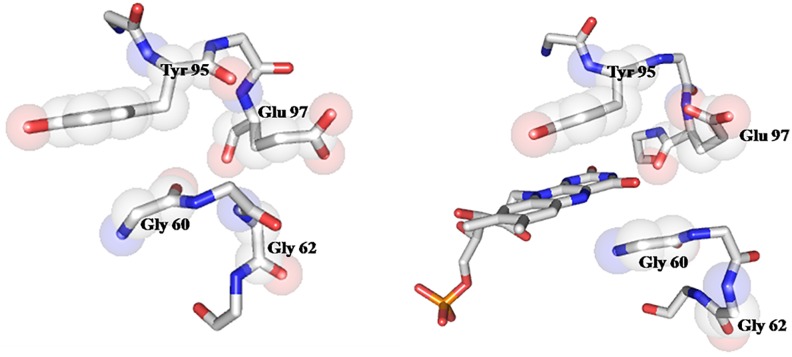
Stick representation of the FMN binding site in the *Sp* apo and holoflavodoxins indicating two key glycine residues (G60 and G62) that close the gap left by FMN in the apoprotein by interacting with residues of the 90’ loop: Y95 and E97.

**Fig 9:**
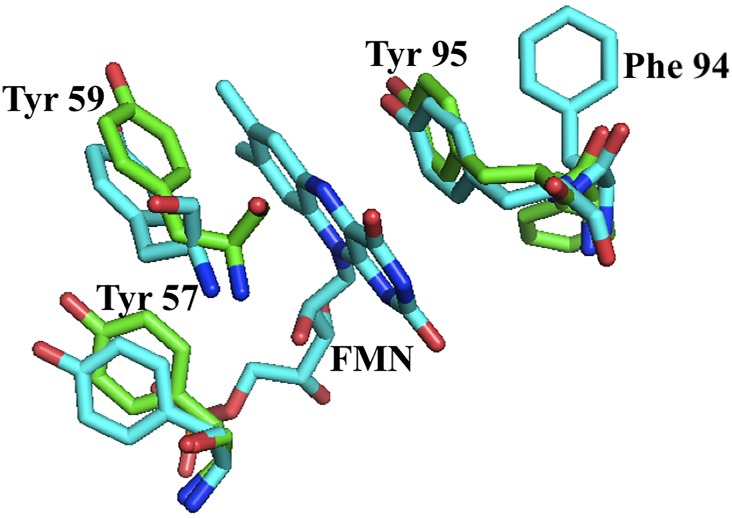
Stick representation of the aromatic/aromatic pairs established by the FMN sandwiching Y59 and Y95 residues with neighboring Y57 and F94, respectively.

Accommodation of the FMN into the *Sp* apoprotein takes place with changes in the dihedral angles of several residues of the isoalloxazine binding loops. In the 57–66 loop, the main changes are observed in residues G60, D61, G62 and E63, while in the 92–100 loop they occur in residues D92, Y95 and D96. No significant changes are noticed in the FMN phosphate binding loop, possibly reflecting that the conformation adopted by this loop in the apoprotein is highly influenced by the phosphate anion bound, which mimics the equivalent moiety in the FMN of the holoprotein. In its bound conformation, the FMN phosphate group forms hydrogen bonds with S10 (sc), M11 (N), T12 (sc and N), N14 (N) and T15 (sc and N) (Fig 6d). As previously reported for the apo/holo flavodoxin pairs in *Anabaena* PCC7119 and *H*. *pylori* [17, 26, 35, 49], the entrance of the FMN cofactor makes the two isoalloxazine binding loops to move apart. In this reorganization, existing interactions between the two loops in the apoform (Y95/G60 and E97/G62) are broken (Fig 8) and the protein expands: the holoprotein surface area is 7745 Å compared to 7135 Å for the apoprotein (S3 Fig). In the holoprotein, FMN is embedded by Y59, D92, E97, Y95, C99 and G60. The isoalloxazine ring of FMN establishes π-π interactions with Y59 and Y95 (Fig 9). While the presence of a FMN binding tyrosine residue is most common in the 90´s-Loop of flavodoxins, in the 50´s-Loop several other residues have been reported (e.g. W, A, M or L).

Interestingly, both Y59 and Y95 establish T-shape interactions with neighboring Y57 and F94, respectively (Fig 9). These aromatic pairs are not present in the apo form and it is possible that they contribute to stabilize the conformation of the FMN-sandwiching residues. No such aromatic pairs have been previously observed in the FMN binding sites of the other short-chain flavodoxins of known structure (e.g. *Desulfovibrio desulfuricans*, *D*. *vulgaris*, *D*. *gigas*, *Clostridium Beijerinckii*, *Escherichia coli YqcA flavodoxin* or *Megasphaera elsdenii*).

**Fig 10:**
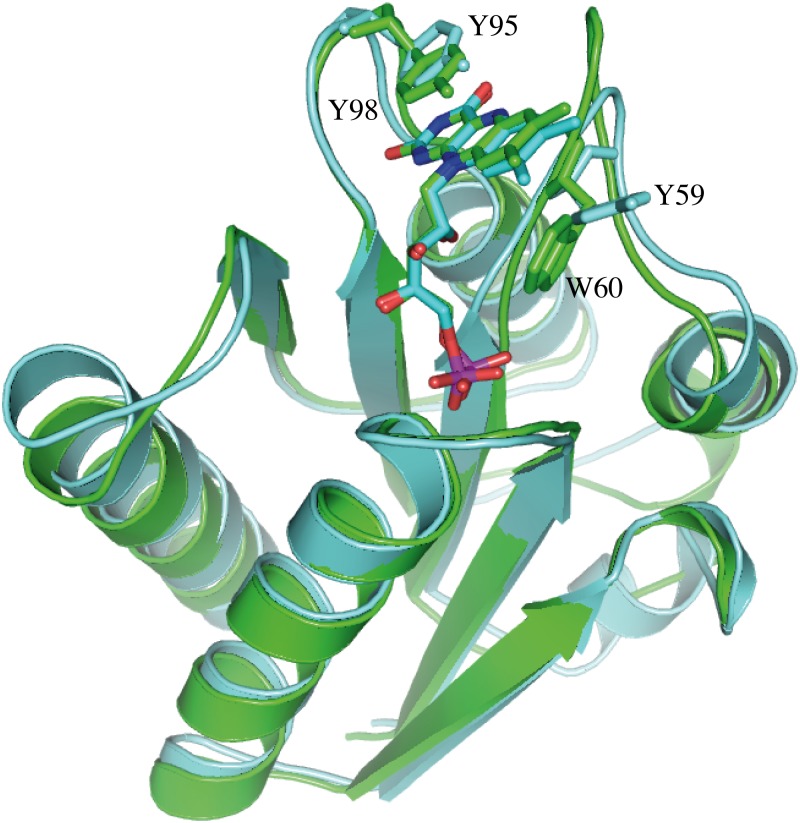
Structural comparison of the short flavodoxins from *Desulfovibrio vulgari*s (pdb id: 1j8q; ribbon and residues in green) and from *Streptococcus pneumoniae* (this work, pdb id: 5lji; ribbon and residues in cyan). The FMN cofactors of the two flavodoxins and the corresponding sandwiching aromatic residues (Y98 and W60 in Dv flavodoxin, and Y59 and Y95 in Sp flavodoxin) are shown in a stick representation.

The flavodoxin from *Desulfovibrio vulgaris* (Dv) is the short-chain flavodoxin for which more detailed structural studies are available. Its sequence shows a 44% identity with that of Sp flavodoxin. The structures of the two flavodoxins (Dv and Sp), superimposed in Fig 10, reveal their high similarity (r.m.s.d. 1.2 Å), the main difference being noticed in the 50´s-Loop, which is longer in Dv flavodoxin. While in the 90´s-Loop both flavodoxins use tyrosine residues with essentially identical orientations on the *si* face of FMN, in the 50´s-Loop the sandwiching aromatic residues in the two flavodoxins are different as are their orientations on the *re* face. The interaction of the FMN phosphate at its binding loop is almost identical in the two flavodoxins, as expected from the high conservation of this loop sequence. Finally, the hydrogen bonding pattern of the polar atoms of the isoalloxazine rings (see Fig 6d for the Sp flavodoxin pattern) is not identical in the two flavodoxins, but it is clearly analogous specially for the nitrogen atoms (N3 and N10).

### Suitability of *Sp*Fld as a novel drug target to combat Streptococcus pneumoniae infections

*Streptococcus pneumoniae* infections cause more than one million deaths every year, affecting principally young children and older people in developing countries. Appearance and propagation of resistant strains, the relative ineffectiveness of some vaccines for some serotypes, and virulence serotype changing, points towards an urgent search for novel *Sp* drug targets [50]. Antibiotics being the main line of therapy to pneumococcal infections, the main issue in pneumococcal infections management is the rising frequency of antibiotic resistance, principally to penicillin, macrolides and also to fluoroquinolones [51]. In this context, the identification of novel *Sp*-specific antibiotics is an important goal. Interestingly, a genome-wide identification of essential genes in *S*. *pneumoniae* TIGR 4 strain in a murine meningitis model identified three genes: adenylosuccinate synthetase (PurA), ABC transporter, and flavodoxin [9]. Although the specific role of flavodoxin in *S*. *pneumoniae* has not been established, it is clear that *Sp* infectiveness in this experimental meningitis model is severely attenuated in a *Sp*Fld knockout mutant [9]. Taking as a precedent our own discovery of specific inhibitors of the essential flavodoxin from the pathogen *Helicobacter pylori*, which are currently under preclinical evaluation [31, 32], we propose that novel *Sp*-specific antimicrobials can be developed based on inhibiting the activity of *Sp* flavodoxin. It is not obvious that the *Hp* inhibitors previously discovered [31, 32] should be effective against *Sp* because *Sp*Fld lacks the pocket near the FMN that is present in *Hp* flavodoxin where an alanine residue replaces the usual aromatic residue at the 50´s-Loop. Therefore, to facilitate the development of *Sp* specific flavodoxin inhibitors, we have cloned, expressed *Sp* flavodoxin, and solved the x-ray structure of both the apoprotein and the holo-form in complex with the FMN cofactor. Besides, we have characterized its thermal stability, a convenient reporting property than can be used to identify potential inhibitors by detecting their stabilizing effects against thermal denaturation exerted upon binding to the target protein. This work provides therefore the missing structural and thermodynamic knowledge of the *Sp* flavodoxin that will facilitate the search for novel antimicrobials to inhibit *Sp*Fld function as a means to control the *Streptococcus pneumoniae* infection.

## Materials and Methods

### Cloning

The flavodoxin coding sequence was amplified from *Streptococcus pneumoniae* TIRGR4 genomic DNA using the following primers:

5´-CGGAATTCGGATCCGGCAGCAGCCATCATCATCATCATCATCATCATGGCAGCGGCCTGGAAGTTCTGTTCCAGGGGCCCATGGCATTAGCAAAAATTG-3´ and 5’-CGGAATTCGTCGACTTAACCCACTTTCGCTGCCAGTTCTTC-3’, which additionally served to introduce 8 His residues at the N-terminal of the expressed protein. The resulting product from amplification was digested and cloned into the pMALC2X using BamHI and SalI restriction sites (underlined). The plasmid was then introduced into DH5alpha *E*. *coli* cells (Life Technologies, Inc., Rockville, MD, USA) and transformed cells were selected on Luria Bertani plates or in liquid medium (LB) containing 100 μg ml^-1^ ampicillin (Sigma, St. Louis, MO, USA). Positive clones identified by colony–PCR (BIORON) were sequenced and shown to contain the DNA construct encoding the full length *Sp*Fld plus 8 additional histidine residues at the *N*-terminus.

### *Sp* flavodoxin expression, purification and apoflavodoxin preparation

The BL21 (DE3) strain (Novagen), cultured in 2XTY media, was used for recombinant protein overexpression. *E*. *coli* BL21 (DE3) cells were grown to an optical density of 0.6–0.8 at 37°C, followed by induction with 1 mM IPTG (Sigma Aldrich). After 10 h of induction at 20°C, the cells were collected by centrifugation at 11295 g for 20 min. The resulting pellet was suspended in 160 ml of binding buffer (150 mM NaCl, 10 mM imidazole, 20 mM sodium phosphate buffer, pH 7.4) per L of culture, and sonicated in ice in the presence of excess FMN, 5 μl of benzonase (Sigma Aldrich, stock solution at 250 units μl^-1^) per 25 ml of solution, lysozyme (Sigma Aldrich, >95% pure) final concentration 1 mg ml^-1^ and 500 μL protease inhibitors cocktail stock solution (1 M PMSF + 10 mM benzamidine + 0.5 mM leupeptin) per 25 ml of cell volume, using 10 cycles, each consisting of a 30 s pulse plus a 30 s cooling step. The lysate was centrifuged at 39086 g for 20 min. The cell pellet and its initial lysate showed a characteristic blue color indicating the flavodoxin FMN cofactor was in the semiquinone redox state [2]. The color changed to yellow (corresponding to oxidized FMN) as the purification progressed.

The supernatant was filtered through a 0.45 μm membrane and loaded onto a Co^2+^HiTrap TALON crude column (GE Healthcare) equilibrated with binding buffer. The column was washed with binding buffer till negative Bradford reaction of the eluate, and the bound protein was eluted using a 150 mM NaCl, 500 mM imidazole, sodium phosphate buffer, pH 7.4 (elution buffer). The eluted solution containing recombinant *Sp*Fld was dialyzed against 150 mM NaCl, 25 mM Tris buffer pH 7.5 to remove the imidazole, and incubated overnight at 4°C with 0.5U of PreScission protease in order to remove the pMAL-(His)8 tail. The mixture was loaded onto coupled GST and histidine columns (GE Healthcare) equilibrated in the dialysis buffer, and *Sp*Fld without affinity tags was collected as the unbinding fraction, dialyzed against 25 mM Tris pH 7.0, loaded into a 5 ml HiTrap Q FF column (GE Healthcare) equilibrated with 5 column volumes in the same buffer, and eluted with 5–10 column volumes with a linear NaCl gradient from 0 to 1M in Tris 25 mM pH 7.0. Pure fractions according to SDS-PAGE were concentrated to 2.5 ml by ultrafiltration using 10 kDa Centricons (Millipore) and loaded onto a gel filtration column (Superdex 200 GE Healthcare) equilibrated with 150 mM NaCl, 25 mM Tris, pH 7.5 from where a single peak corresponding to the monomer was collected. The purity of the obtained *Sp*Fld was estimated to be >95% by SDS/PAGE (S4 Fig). A single band of 16 kDa was observed, in agreement with the expected molecular mass of 16025 Da. *Sp*Fld was dialyzed against 150 mM NaCl, 100 mM MES pH 6.5, and concentrated to around 1 mM. Aliquots were fast frozen in liquid nitrogen and stored at -80°C for further use.

For the crystallization of FMN-bound flavodoxin, selenomethionine substituted *Sp*Fld was similarly expressed in *E*. *coli* BL21 (DE3) strain, using the SelenoMethionine media from Molecular Dimensions. The protein was purified following the same protocol used for the native protein (see above). MALDI-TOF mass spectrometry, carried out in the Proteomics facility from UAB, showed that three out of four possible selenomethionine residues were incorporated into the protein. *Sp* apoflavodoxin (deprived from the FMN cofactor) was obtained by precipitation with trichloroacetic acid (TCA) [52]. The protein pellet was washed five times with 3% w/v TCA and 1 mM DTT, resuspended in 1 M MES pH 6.5, 15 mM NaCl, and subsequently dialyzed in 150 mM NaCl, 100 mM MES, pH 6.5 and stored as the holoprotein.

### UV-Visible absorption, circular dichroism and fluorescence emission spectra

The UV-visible absorption spectrum of holo and apo *Sp*Fld in 150 mM NaCl, 100 mM MES, pH 6.5, was collected at 25°C using a Cary 100Biospectrometer (Varian). To estimate *Sp*Fld apoflavodoxin molar extinction coefficient in 150 mM NaCl, 100 mM MES, pH 6.5 (working buffer), the Gill von Hippel method was used [37]. The extinction coefficient of the denatured protein in 6 M guanidine-HCl, 20 mM phosphate sodium, pH 6.5, was first calculated from its content of cysteines and tyrosines. Then, the ratio of absorbances at the wavelength selected of protein solutions or equal concentration prepared in either denaturing buffer or in working buffer equaled the ratio of the calculated extinction coefficients in denaturing buffer over that in working buffer. All measures were done by triplicate. To determine the holoflavodoxin extinction coefficient, the Mayhew and Massey method was used [38].

Far-UV, near-UV and visible-UV circular dichroism (CD) spectra of the holo and apoproteins in 150 mM NaCl, 100 mM MES, pH 6.5, were acquired at 4°C in a Chirascan spectropolarimeter (Applied-Photophysics). For near-UV and visible spectra, 80 μM protein solutions in a 1-cm path-length cuvette were used, while for far-UV, 80 μM protein solutions were used in a 0.01-cm path-length cuvette. To accurately determine the concentration of protein in the CD samples, small volumes of concentrated stock solutions of either apo or holoflavodoxin were initially diluted to prepare solutions that provided an approximation to the actual concentrations. Based on that, protein samples around 80 μM were prepared and used to acquire the CD spectra. After having recorded the CD spectra, the actual concentrations of those samples were accurately determined by recording their full absorbance spectra. For apoflavodoxin quantification the near-UV peak was used, and for holoflavodoxin quantification the major visible peak was used. The absorbances of either of those peaks were of around 0.7 in a 1 cm path-length cuvette and allowed a fine determination of protein concentration using the corresponding extinction coefficients. These concentrations were finally used for the MRE calculation leading to Fig 2a. Fluorescence emission spectra of 20 μM holoprotein or apoprotein, in 150 mM NaCl, 100 mM MES, pH 6.5, were recorded at 10°C from 275 to 750 nm or from 290 to 500 nm, respectively, with excitation at 270 nm, using a Cary Eclipse spectrofluorometer (Varian).

### Thermal denaturation followed by spectroscopy

Holo and apo *Sp*Fld thermal denaturation was followed, from 8 to 90°C, by CD. Unfolding curves for the holoprotein were acquired at 220 nm (far-UV CD) and at 272 nm (near-UV CD) with heating rates of 1°C min^-1^. For the apoprotein, the wavelengths used were 221 and 285 nm, respectively. For near-UV, a 1-cm path-length quartz cuvette was used and the samples were prepared with the same buffer and protein concentration used for recording the CD spectra, while for far-UV CD a 0.1-cm path-length cuvette and 80 μM protein solutions were used. The thermal denaturation of the holoprotein was followed, from 8 to 80°C, by fluorescence in the visible region (excitation and emission of 464 and 525 nm, respectively) associated to FMN release coupled to protein unfolding, using a heating rate of 1°C min^-1^. Protein concentration and buffer conditions were those used for acquiring the fluorescence spectrum (20 μM for both, holo- and apoprotein). Each individual curve was fitted to a two-state model, as previously described [53, 54], using the following equations:

$$S=\;\frac{{\text{S}}_{\text{N}}{\;+\text{ m}}_{\text{N}}\;\times \text{ T }+\;\left({\text{S}}_{\text{U}}{\;+\text{ m}}_{\text{U}}\;\times \text{ T}\right){\;\times \text{ e}}^{\text{-}\Delta G/\text{RT}}}{{\text{1}+\text{e}}^{\text{-}\Delta G/\text{RT}}}$$ (1)

$$\Delta G\left(\text{T}\right)=\Delta H_{\text{m}}\times \left(1-\left(\text{T}/{\text{T}}_{\text{m}}\right)\right)-\Delta {\text{C}}_{\text{P},\text{m}}\times \left(\left({\text{T}}_{\text{m}}-\text{T}\right)+\text{T}\times \text{ln}\left(\text{T}/{\text{T}}_{\text{m}}\right)\right)$$ (2)

where “*S”* is the observed spectroscopic signal at a given temperature, and “S_N(U)_ + m_N(U)_ × T” represents the temperature-dependent intrinsic signal of either the native (N subscript) or the unfolded (U subscript) protein, R is the ideal gas constant, T is the absolute temperature, and Δ*G* is the unfolding Gibbs energy change, which can be expressed in terms of the mid-transition temperature T_m_, the unfolding enthalpy change Δ*H*_m_, and the unfolding heat capacity change Δ*C*_P,m_. Because all the individual thermal unfolding curves of either the apo- or the holoprotein acquired following different spectroscopic signals could be superimposed, each group of curves (from either holo or apoflavodoxin) was also globally fitted to the two-state unfolding model, as described [53, 54].

### Thermal denaturation followed by differential scanning calorimetry

Differential scanning calorimetry (DSC) assays were performed on 40 μM apo *Sp*Fld in the absence or presence of FMN (40 and 1000 μM). Measurements (from 10 to 100°C at a scanning rate of 1°C min^-1^) were performed in a VP-DSC microcalorimeter (MicroCal LLC, Northampton, MA), and rescans were recorded after cooling to 10°C. The reference and sample solutions were degassed and carefully loaded into the cells to avoid bubble formation. Baselines were recorded before each assay with the reference and sample cells filled with buffer (150 mM NaCl, 100 mM MES, pH 6.5). Data analysis, using the software package ORIGIN (OriginLab), was based on the model-free van´t Hoff analysis. Briefly, from the thermal unfolding assay the mid-transition temperature *T*_*m*_ and the calorimetric enthalpy Δ*H*_cal_ could be determined and, from those values, the van’t Hoff enthalpy was estimated as:

$$\Delta H_{\text{vH}}=\frac{4{\text{RT}}_{\text{m}}^{2}{\langle \Delta {\text{C}}_{\text{P},\text{tr}}\rangle }_{\text{max}}}{\Delta {\text{H}}_{\text{cal}}}\;$$ (3)

where <ΔC_P,tr_>_max_ is the maximal value of the excess molar heat capacity of the protein [55]. The van’t Hoff enthalpy is equivalent to the unfolding enthalpy obtained from analysis of spectroscopic techniques, and: a) it is equal to the calorimetric enthalpy if the thermal unfolding follows a two-state mechanism; b) it is smaller than the calorimetric enthalpy if the thermal unfolding follows a non-two-state mechanism with partially folded intermediates; and c) it is higher than the calorimetric enthalpy if the thermal unfolding follows a two-state mechanism where the native protein self-associates in oligomers [56]. Once the appropriate unfolding model has been determined, the unfolding curve can be analyzed accordingly.

### FMN binding by isothermal titration calorimetry

Isothermal titration calorimetry (ITC) measurements were carried out in a MicroCal Auto-iTC200 (MicroCal/GE Healthcare) using carefully degassed cofactor and protein solutions. A 300 μM solution of FMN (Sigma-Aldrich, >95% pure) dissolved in 150 mM NaCl, 100 mM MES, pH 6.5 was titrated into 20 μM apo *Sp*Fld, in the same buffer. Intervals of 700 s between injections were allowed to guarantee recovery of the base line. Data were analyzed with a one-site binding model implemented in the Origin (OriginLab) software package. Binding titrations were performed at 15, 20 and 25°C to obtain the binding heat capacity as the temperature derivative of the observed binding enthalpy. Additional assays required for assessing proton exchange processes coupled to ligand binding were performed in 150 mM NaCl 50 mM MOPS pH 6.5 at 25°C. The buffer-independent binding enthalpy Δ*H*_0,_ and the net number of protons exchanged n_H_ were estimated by performing assays using buffers with different ionization enthalpies and linear regression using eq 4:

$$\Delta H_{obs}=\Delta H_{0}+n_{H}\Delta H_{\text{ion}}$$ (4)

where Δ*H*_*obs*_ is the enthalpy obtained in a given assay and Δ*H*_*ion*_ is the ionization enthalpy of the buffer.

### Dithionite titration of *Sp*Fld holoflavodoxin

To observe the spectra of the different redox states of *Sp*Fld, a sodium dithionite reduction was performed at 10°C using 20 μM protein in 150 mM NaCl, 100mM MES buffer, pH 6.5 under pseudo-anaerobic conditions. A concentrated anaerobic sodium dithionite solution (50 mM) was prepared in the same buffer by sequential evacuation and re-equilibration with oxygen-free argon. Pseudo-anaerobic conditions were achieved in the spectrophotometer cell by argon flux. Stepwise *SpFld* reduction was achieved by addition of 2.5–10 ml aliquots of sodium dithionite to the protein sample. After each addition, the UV-Vis spectrum was recorded in a Cary 100 spectrophotometer (Varian).

### Crystallization and data collection

Crystallization trials were carried out using the sitting-drop vapor diffusion method at 18°C. The drops were prepared by mixing 0.5 μL of protein solution containing either holo (62 mg ml^-1^) or apoprotein (127 mg ml^-1^) in 100 mM MES, 150 NaCl, pH 6,5 buffer with 0.5 μL of the mother liquor. Crystals of the holoprotein (though these crystals contain selenomethionine, they are referred here as our holo form) were grown in 45% 2-Methyl-2,4-pentanediol (MPD), 100 mM Bis-Tris pH 5.5 and 200 mM calcium chloride, apoprotein form crystals were obtained in 10% glycerol and 2 M ammonium sulphate. Crystals were formed after 3–8 days. Best apoprotein and holoprotein *Sp*Fld crystals were immersed, respectively, in the same reservoir solution or in the same solution with 20% of glycerol for cryoprotection. All diffraction datasets were collected at the Diamond synchrotron light source (DLS).

### Structure determination, phase calculation, model building and refinement

Initial molecular replacement trials were attempted using MOLREP [57] with the data from a holo crystal processed as H3 (note that the holo form contains SeMet residues) and the structure of *Desulfovibrio desulfuricans* flavodoxin (PDB code: 3F9O) as search model; however, no improvement in R_factor_ and R_free_ was observed during refinement. The program SOLVE [58] was used for initial phasing by single anomalous dispersion (SAD). Despite the data collection had been performed using a suboptimal wavelength (λ = 0.976 **Å**), the positions of the 3 Se atoms were determined. Subsequent phasing and solvent flattening were carried out using SOLVE and RESOLVE [59] as implemented in PHENIX [60]. A solvent flattened Se-SAD derived electron density map was used for automatic construction of the holo *Sp*Fld structure with RESOLVE. The automatic building process resulted in a model formed by 76 residues in 11 chains. Remarkably, one of the chains was an alpha helix that appeared at the same position in the previous MR attempt. Then, we built a more complete model with the program *PHENIXAutoBuild* and using the initial MR solution as initial model in combination with the Se-SAD density map obtained previously. The resulting model contained 143 residues positioned in 2 chains, with R_fact_ and R_free_ of 0.29 and 0.36 respectively. We completed the model manually with Coot [61] and refined with REFMAC [62], obtaining R_fact_ and R_free_ values of 0.25 and 0.33 respectively. Finally, the combination of adding FMN, water molecules and new rounds of model building in Coot and TLS restrained refinement in REFMAC, rendered R_fact_ and R_free_ values of 0.187 and 0.228, respectively.

To solve the structure of the *Sp*Fld apoprotein, molecular replacement was performed using MOLREP[58] with the holoprotein *Sp*Fld structure as starting model and the data in the space group P2_1_2_1_2_1_. Iterative rounds of manual building in Coot and TLS restrained refinement in REFMAC were also performed, and the final model was validated with PROCHEK [63]. All figures were generated with PyMoL (http://wwwrcsb.org). Coordinates and structure factors have been deposited in the protein data bank under accession code 5LJI and 5LJL for the holo and apo form, respectively.

## Supporting Information

S1 Fig Electron density map is F_O_ − F_C_ syntheses (blue) contoured at 2.2 s for FMN. (PNG)

S2 Fig Superimposition of apo *Sp*Fld (blue) and sulfate anion (magenta-red) with apo flavodoxin structures of *E*. *coli* MioC (pale blue) (a), *Helicobacter pylori* (yellow) and chloride anion (green) (b), *Anabaena* (salmon) and sulfate ion (c). (TIF)

S3 Fig Surface representation of apo (a) and holo form (b) of *Sp*Fld. Protein is coloured in grey whereas FMN carbon atoms are coloured in white. Note that FMN induces the formation of a larger pocket in the holo form (in which FMN locates) in comparison with a smaller pocket present in the apo structure.
(TIF)

S4 Fig *Sp*Fld size and purity analyses by 12% SDS-PAGE; line 1: molecular mass markers, line 2: *Sp*Fld at the end purification process. (TIF)
