# Supplementary figures and images for: Streptococcus pneumoniae TIGR4 Flavodoxin: Structural and Biophysical Characterization of a Novel Drug Target
Source: PLoS One. 2016 Sep 20;11(9):e0161020. doi: 10.1371/journal.pone.0161020 (PMC5029806; doi:10.1371/journal.pone.0161020)

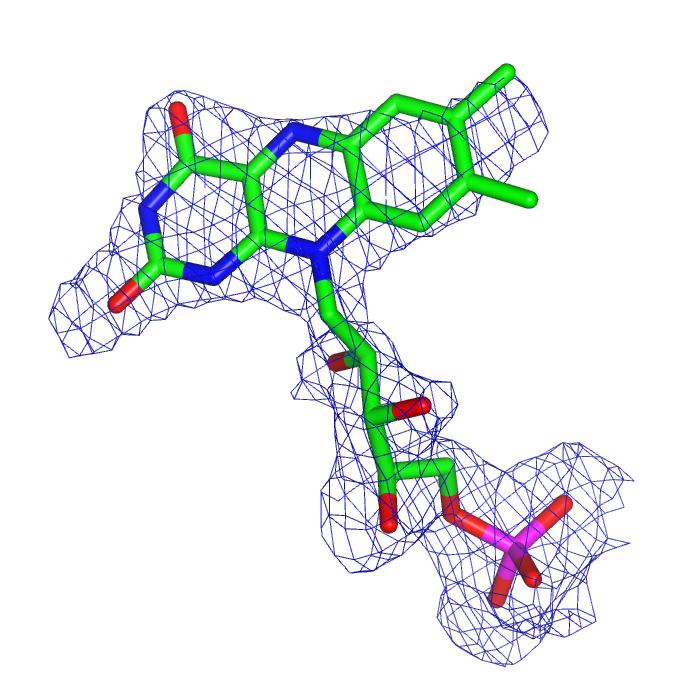

Supplement: S1 Fig — (PNG) [file pone.0161020.s001.png]

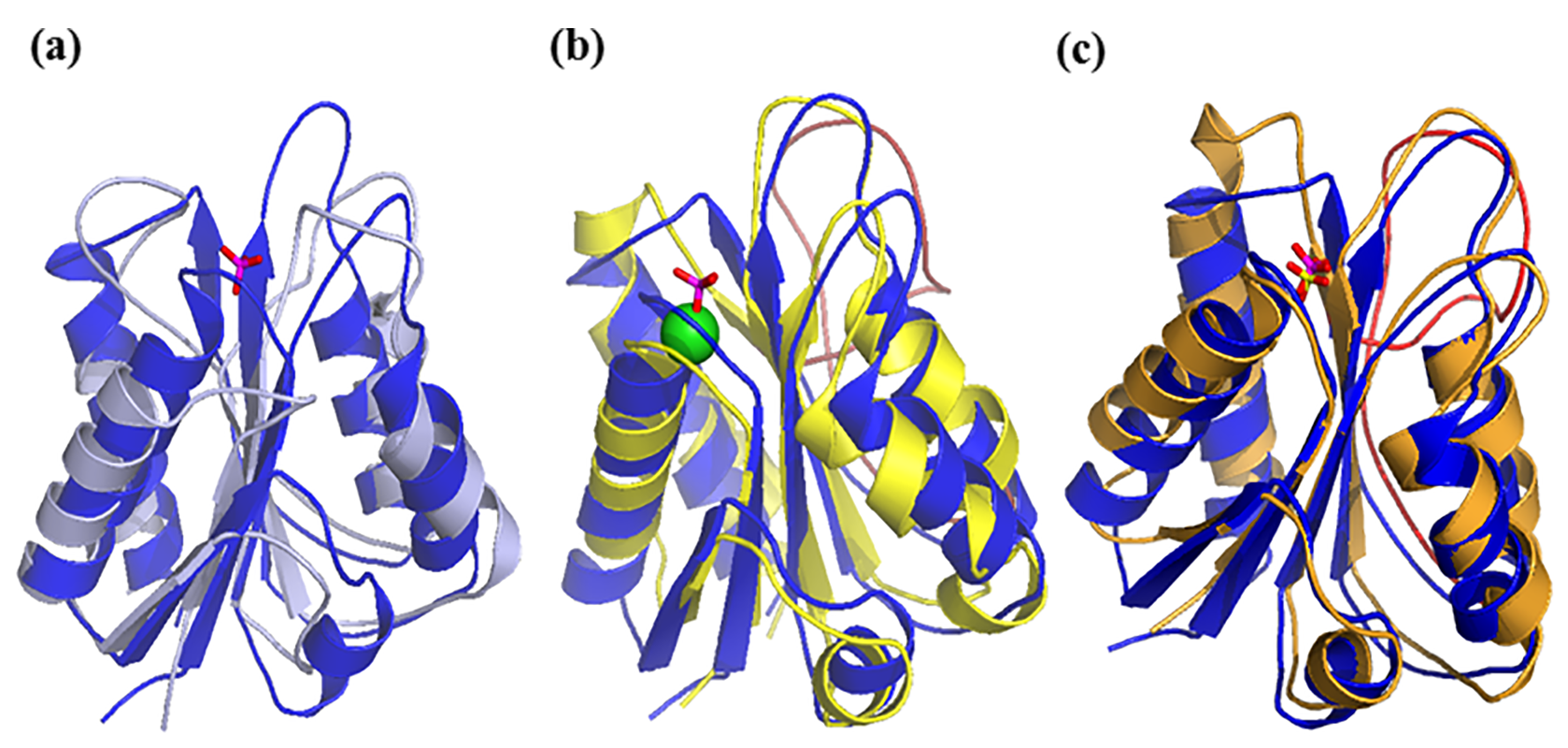

Supplement: S2 Fig — (TIF) [file pone.0161020.s002.tif]

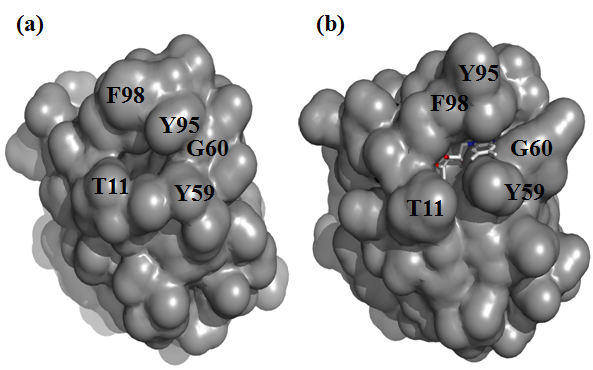

Supplement: S3 Fig — Note that FMN induces the formation of a larger pocket in the holo form (in which FMN locates) in comparison with a smaller pocket present in the apo structure. (TIF) [file pone.0161020.s003.tif]

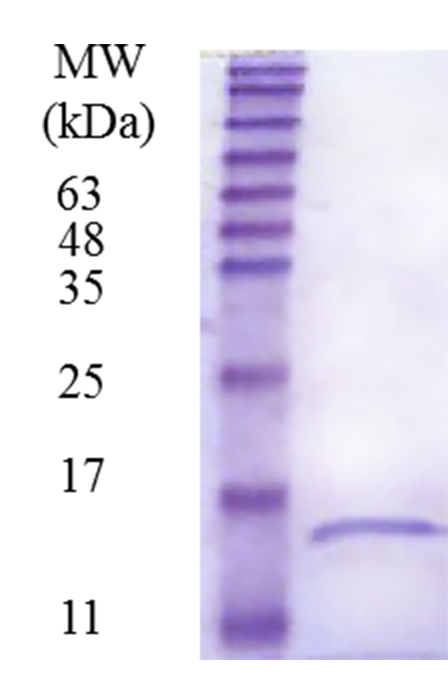

Supplement: S4 Fig — (TIF) [file pone.0161020.s004.tif]
